# Integrated Analysis of a Gene Correlation Network Identifies Critical Regulation of Fibrosis by lncRNAs and TFs in Idiopathic Pulmonary Fibrosis

**DOI:** 10.1155/2020/6537462

**Published:** 2020-06-02

**Authors:** Fan Wang, Pei Li, Feng-sen Li

**Affiliations:** ^1^Xinjiang Medical University, Urumqi, Xinjiang 830000, China; ^2^Kelamayi City Dushanzi People's Hospital, Xinjiang 830000, China; ^3^Traditional Chinese Medicine Hospital Affiliated to Xinjiang Medical University & National Clinical Research Base of Traditional Chinese Medicine, Urumqi, Xinjiang 830000, China

## Abstract

Idiopathic pulmonary fibrosis (IPF), the most frequent form of irreversible interstitial pneumonia with unknown etiology, is characterized by massive remodeling of lung architecture and followed by progressive loss of lung function. However, the key regulatory genes and the specific signaling pathways involved in the onset and progression of IPF still remain unclear. The present study is aimed at investigating the key role of long noncoding RNAs (lncRNAs) and transcription factors (TFs) involved in the pathogenesis of IPF through the integrated analysis of three gene expression profiles from the GEO dataset (GSE2052, GSE44723, and GSE24206). A total of 8483 differentially expressed genes (DEGs) including 988 upregulated and 7495 downregulated genes were filtered. Subsequently, following the intersection of these DEGs, 29 overlapping genes were identified and further analyzed using a bioinformatics approach. Furthermore, the protein-protein interaction (PPI) network was used to obtain 18 modules of related genes. The hub genes were identified through hypergeometric testing, which were closely associated with ubiquitin-mediated proteolysis, the spliceosome, and the cell cycle. The significant difference was observed in the expression of these key genes, such as lncRNA MALAT1, E2F1, and YBX1, in the peripheral blood of IPF patients when compared with those normal control subjects by real-time polymerase chain reaction (RT-PCR) analysis. This study indicated that *lncRNA MALAT1*, *E2F1*, and *YBX1* may be key regulators for the pathogenesis of IPF.

## 1. Introduction

Idiopathic pulmonary fibrosis is a chronic and progressive lung tissue damage of unknown etiology, which is characterized by the abnormal proliferation of activated fibroblasts/myofibroblasts and excessive deposition of collagen in the extracellular matrix (ECM) from adjacent alveoli to the lung parenchyma. IPF has a poor prognosis and high mortality rate with the postdiagnosis median survival rate of only 20% to 30% and the median survival of approximately 3 to 5 years [1, 2]. Due to the complexity and heterogeneity of IPF, its incidence and mortality rate, which has a positive relationship with advanced age, have shown a steadily increasing trend worldwide [3]. Although the pharmacotherapy of IPF has made certain progress over the past 5 years, the therapeutic efficacy is unsatisfactory because of the variable and unpredictable course of IPF and large individual differences [4].

Increasing studies related to transcriptome, including both protein-coding mRNAs and noncoding RNAs (ncRNAs), have provided novel insights into the molecular mechanism of IPF pathogenesis. Among them, ncRNAs implicated in multiple fibrotic diseases have been divided into short and long ncRNAs (lncRNAs) based on its length of nucleotide sequences. Multiple studies have shown that lncRNAs (≥200 nucleotides) contribute to the pathogenesis and progression of IPF and gain more attention [5, 6]. However, varying proportions of transcripts that can be detected and the accuracy of measurements of changes in low-abundance transcripts reduce detection accuracy of lncRNAs in transcriptome-related lung fibrosis research. In addition, transcripts detected and measured are very different in different microarray platforms. These factors imply that some lncRNAs may be overlooked and false-positive or false-negative results may be generated [7, 8]. Based on publicly available microarray expression datasets in the Gene Expression Omnibus (GEO) database, an in-depth bioinformatics analysis of lncRNAs may provide a comprehensive understanding of not only transcriptional regulation but also posttranscriptional regulation. Hence, using bioinformatics methods to analyze the comprehensive gene network, this study was performed to identify the biological processes and pathways of differentially expressed genes (DEGs) that are involved in the pathogenic mechanism of IPF. These results may be useful in elucidating the critical regulatory mechanism of IPF from a systematic perspective and providing the relevant effective interventions to attenuate or reverse the process of lung fibrosis.

## 2. Materials and Methods

### 2.1. Microarray Data Information

NCBI-GEO (http://www.ncbi.nlm.nih.gov/geo/) is a free database repository comprising microarray/gene profile, next-generation sequencing, hybridization array, and chip data. All data were derived from GEO datasets GSE2052, GSE44723, and GSE24206. The microarray data of GSE2052 were based on GPL1739 Platforms (Amersham Biosciences CodeLink Uniset Human I Bioarray, University of Pittsburgh, PA, USA) and included 15 IPF and 11 control lung tissues (submission date: 09 December 2004) [9, 10]. The GSE44723 data were based on GPL570 Platforms (Affymetrix Human Genome U133 Plus 2.0 Array, Affymetrix, Santa Clara, CA, USA) and included 10 pulmonary fibrosis and 4 normal lung tissues (submission date: 10 April 2013) [11]. The GSE24206 data were based on GPL570 Platforms (Affymetrix Human Genome U133 Plus 2.0 Array, Affymetrix, Santa Clara, CA, USA) and included 17 IPF and 6 normal lung tissues (submission date: 01 November 2011) [12]. The total RNA of the samples was extracted to analyze the genomic profile of the RNA. All data came from expression profiling with microarrays conducted for *Homo sapiens*.

### 2.2. Identification of Differential Gene Expression in IPF

The original data from these datasets, including SOFT-formatted family files and Series Matrix Files, were downloaded for analysis. DEGs were identified with the R package limma (http://bioconductor.org/packages/release/bioc/html/limma.html). Unsupervised hierarchical clustering was performed to center the normalized and log2-scaled expression values on the median by using Cluster 3.0 (Fig. S1–S3). After pretreatment of the genes that came from more than one probe set, the DEGs identified with cutoff criteria of |logFC| > 1 and *P* < 0.05 by the classic *t*-test were considered statistically significant.

### 2.3. Gene Ontology and KEGG Enrichment Analysis of DEGs

Functional and pathway enrichment analyses of candidate DEGs were performed with the online bioinformatics Database for Annotation, Visualization, and Integrated Discovery (DAVID, http://david.ncifcrf.gov) (version 6.7), which can integrate biological data and comprehensively annotate the biological functional information of genes. Gene Ontology (GO) analysis can provide annotation of DEGs regarding biological processes (BPs), molecular functions (MFs), and cellular components (CCs) and allows further analysis of the bioprocesses of these genes. The Kyoto Encyclopedia of Genes and Genomes (KEGG) provides high-level functions and biological system information derived from large-scale molecular datasets generated with high-throughput experimental technologies. DAVID was applied to analyze the function of DEGs, and a *P* value of less than 0.05 was considered statistically significant.

### 2.4. Construction of Protein-Protein Interaction (PPI) Network and Module Analysis

Functional analysis of interactions between the candidate DEG-encoded proteins can provide a new perspective on the pathogenesis and development of IPF. The protein-protein interaction network (PPI) of DEGs was constructed with the Search Tool for the Retrieval of Interacting Genes (STRING) online database (http://string-db.org) (version 11.0) considering combined scores of interaction greater than 0.4 to indicate statistical significance, and the network was visualized in the form of modules by using ClusterONE Cytoscape plug-in (version 1.0) [13]. Cytoscape (version 3.6.1) is powerful bioinformatics software that is utilized to visualize molecular interaction networks. Then, GO and KEGG enrichment analyses of genes in the module were conducted by using DAVID.

### 2.5. Selection of the Key Genes

Using the Molecular Complex Detection (MCODE) (version 1.4.2) plug-in of Cytoscape, the hub genes were selected by means of clustering the dense connection domain based on the topology of a given network [14]. The GO and pathway enrichment analyses of hub genes were performed with the ClueGO (2.5.1) plug-in of Cytoscape [15]. Subsequently, the biological pathway relationship network of these hub genes was constructed with the Biological Networks Gene Ontology tool (BiNGO) (version 3.0.3) plug-in of Cytoscape [16]. Using the hypergeometric test of the empirical Bayes approach, key genes were obtained through calculation. A *P* value of less than 0.05 was considered statistically significant.

### 2.6. Subjects and Blood Samples

Considering the particular and complex nature of IPF, not all patients can undertake the invasion operation including bronchial and surgical lung biopsies to obtain the lung tissue samples. Moreover, obtaining healthy control samples would be not only extremely difficult but also restricted by ethical concerns. Due to the feasibility and convenience of obtaining blood samples, we validated the expression levels of candidate genes in the peripheral blood samples of all subjects. IPF patients (*n* = 20) were diagnosed at the Traditional Chinese Medicine Hospital Affiliated to Xinjiang Medical University. Healthy physical examinees (*n* = 20) were selected as the control group. The cohort of 40 subjects provided written informed consent in compliance with the code of ethics of the World Medical Association. The collection and usage of the blood samples were approved by the Medical Research Ethics Committee of Traditional Chinese Medicine Hospital Affiliated to Xinjiang Medical University (the Scientific Research Project 2018XE0109-1).

### 2.7. Validation of Key Genes by RT-PCR Analysis

Purification of RNA from blood samples from 20 IPF patients and 20 normal control subjects was performed using the TRIzol™ LS Reagent (Invitrogen, USA). The RNA was reverse-transcribed using the PrimeScript™ RT reagent kit with ɡDNA Eraser (TAKARA, Japan) according to the manufacturer's recommendations. The cDNA from each sample was used as a template with GAPDH as an internal reference. The specific primer sequences used to amplify the 4 key candidate genes are listed in Table S1. Real-time PCR (RT-PCR) was performed using the StepOnePlus™ Real-Time PCR System (Thermo Fisher Scientific, USA). The results are represented as the means of 3 repetitions and were quantified via the 2^-*ΔΔ*ct^ method. The mRNA levels of key genes between the IPF and normal lung tissues were compared using a paired *t*-test (*P* < 0.05) using GraphPad 6.0 (GraphPad Software, La Jolla, CA, USA). The data were presented as the mean ± standard deviation$\left(\bar{X}\pm S\right)$ (Table S2). Counting data was assessed using a *χ*^2^ test. Multiple-group comparison was assessed using one-way analysis of variance (ANOVA) followed by the Bonferroni multiple comparison test. Comparison of two groups was assessed by a two-tailed *t*-test.

## 3. Results

### 3.1. Identification of Differentially Expressed Genes in IPF

**Figure 1:**
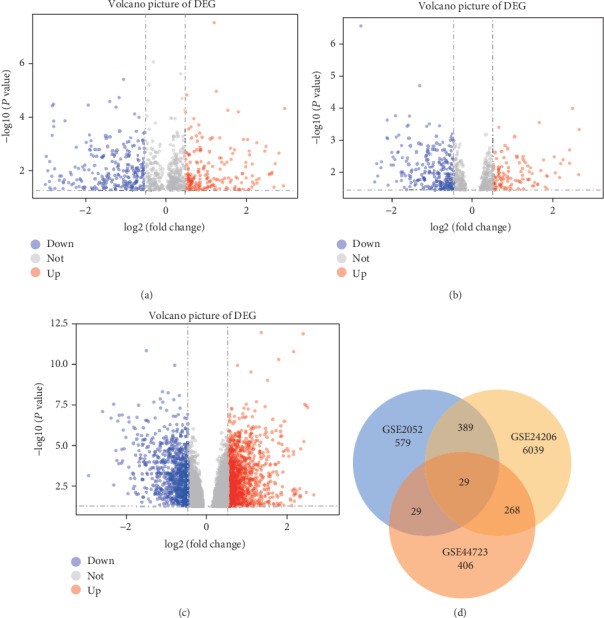
Identification of DEGs from three GEO datasets (GSE2052, GSE44723, and GSE24206). (a–c) Volcano plot of the distributions of DEGs from the three datasets, mapping upregulated genes (red dots) and downregulated genes (blue dots). No significantly changed genes are marked as gray dots. (d) Identification of 29 commonly changed DEGs using Venn diagram from these three datasets (http://www.ehbio.com/ImageGP/index.php/Home/). Differently colored areas represented different datasets. The overlap areas meant the commonly changed DEGs. DEGs were identified with a paired *t*-test. The *P* value of less than 0.05 and [logFC] value of more than 1 were considered statistically significant.

After normalization and standardization of the raw data from these three GSE2052, GSE44723, and GSE24206 datasets (Figures 1(a)–1(c)), we identified a total of 8483 aberrantly expressed genes, including 988 upregulated and 7495 downregulated genes, in IPF tissues compared to normal lung tissues (Table 1). There were 29 overlapping genes between the GSE2052, GSE24206, and GSE44723 datasets according to the Venn diagram, including 29 overlapping genes between GSE2052 and GSE44723, 268 overlapping genes between GSE44723 and GSE24206, and 389 overlapping genes between GSE2052 and GSE24206 (Figure 1(d)).

### 3.2. GO and KEGG Enrichment Analyses of Differentially Expressed Genes

The biological processes associated with the DEGs were determined by using the DAVID online bioinformatics database. As shown in Table 2, the top 6 GO results revealed that the significantly enriched BPs of IPF DEGs were mainly concentrated in the cell adhesion, biological adhesion, regulation of cell proliferation, and so on. The top 6 significantly enriched MFs were mainly concentrated in the calcium ion binding, cytokine binding, chemokine activity, and so on. The top 6 significantly enriched CCs were mainly concentrated in the extracellular region part, extracellular space, extracellular matrix, and so on. The top 6 significantly enriched KEGG pathways were mainly concentrated in the ECM-receptor interaction, cytokine-cytokine receptor interaction, and so on.

### 3.3. Construction and Enrichment Analysis of Modules

**Figure 2:**
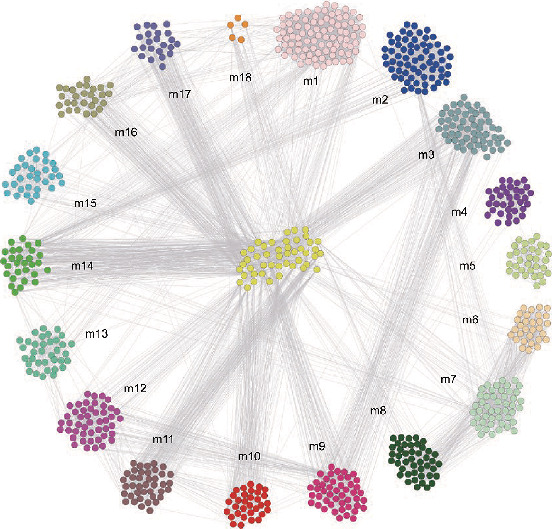
The module genes were filtered into the DEG protein-protein interaction (PPI) network complex.

**Figure 3:**
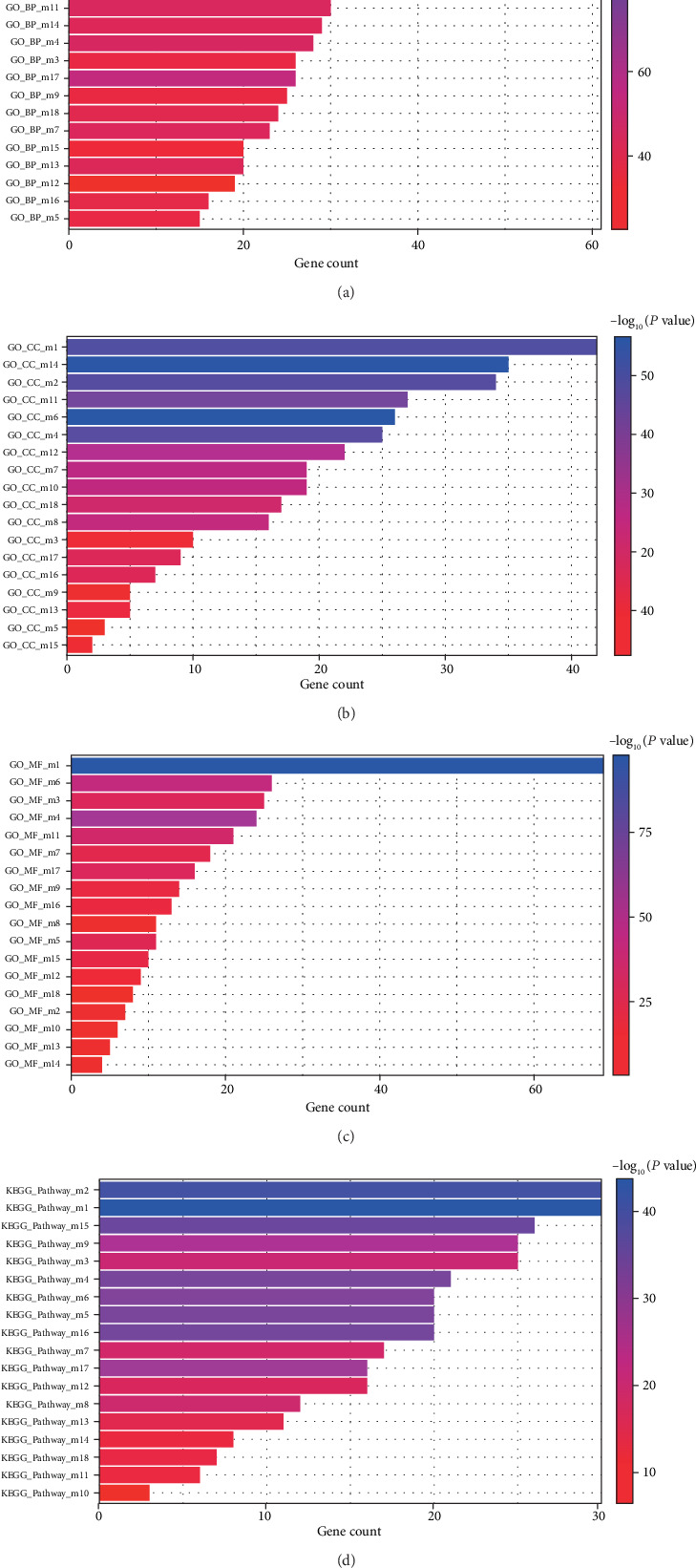
Gene Ontology and KEGG analysis of module gene function in idiopathic pulmonary fibrosis. (a–c) Gene Oncology (GO) analysis was conducted to identify overrepresented GO terms for the significantly enriched analysis of module genes. (d) KEGG analysis was conducted to identify the biological information of module genes.

The IPF DEGs were used to construct the PPI network by using the STRING online database, and a total of 18 modules were obtained with the ClusterONE Cytoscape plug-in (Figure 2). To obtain functional and pathway enrichment information, the genes involved in these 18 modules were further analyzed by using DAVID as shown in Figures 3(a)–3(d). The top 10 modules of significantly enriched BPs were mainly concentrated in the protein polyubiquitination, ciliary basal body-plasma membrane docking, Golgi vesicle transport, and so on. The top 10 modules of significantly enriched CCs were mainly concentrated in the ubiquitin ligase complex, microtubule organizing center part, microtubule-associated complex, and so on. The top 10 modules of significantly enriched MFs were mainly concentrated in the ubiquitin-protein transferase activity, structural constituents of the cytoskeleton, microtubule motor activity, and so on (Table 3). The KEGG pathways of these 18 modules were concentrated in the ubiquitin-mediated proteolysis, spliceosome, purine metabolism, Fanconi anemia pathway, and so on (Table 4).

### 3.4. Selection and Analysis of Key Genes

**Figure 4:**
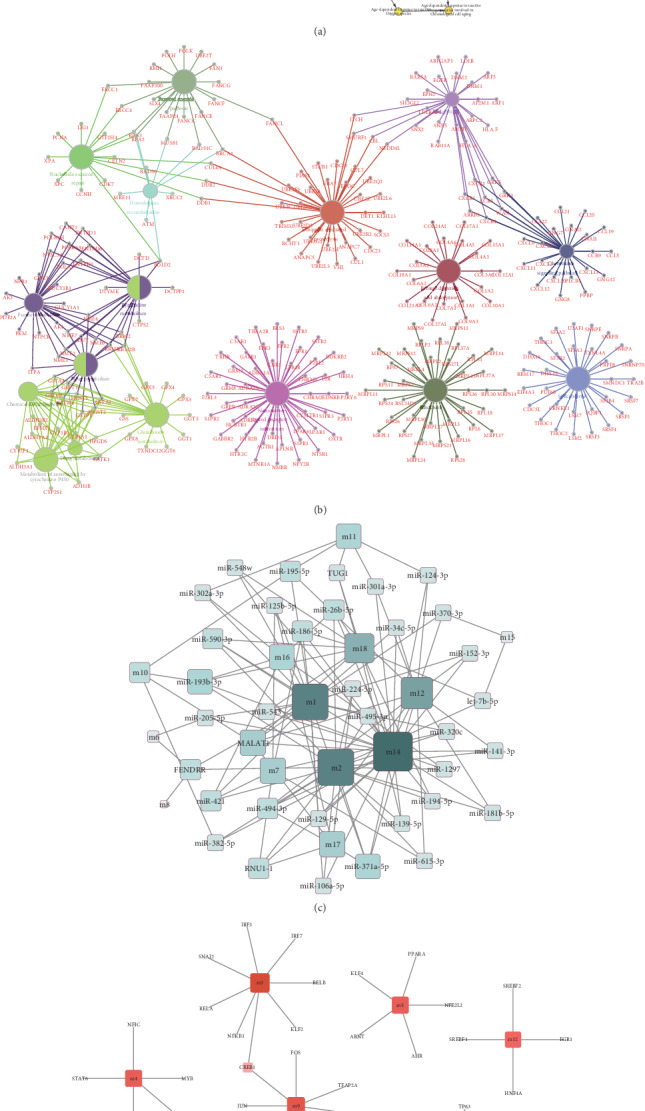
Identification and biological analysis of key genes in idiopathic pulmonary fibrosis. (a) Using BiNGO plug-in of Cytoscape, the biological network of the significant DEGs was conducted. (b) Using ClueGO plug-in of Cytoscape, GO and KEGG analyses were conducted to identify the significant DEGs. (c) The ncRNAs in module genes were identified using the hypergeometric test. (d) The TFs in module genes were identified using the hypergeometric test.

The biological network of differentially expressed IPF genes was constructed by using the BiNGO plug-in of Cytoscape, and the results revealed that most of the DEGs were significantly enriched in mitochondrial translation, cellular macromolecule metabolic process, cellular process, and so on (Figure 4(a)). ClueGO, another plug-in of Cytoscape, can annotate and visualize the pathway networks of DEGs integrating GO terms as well as KEGG pathways. The results from ClueGO revealed that most of the DEGs were significantly enriched in the glutathione metabolism, Fanconi anemia pathway, etc. (Figure 4(b)).

Subsequently, the key genes were obtained through calculation of the hypergeometric test. The 30 miRNAs and 4 lncRNAs enriched in 13 modules and the 44 transcription factors (TFs) enriched in 10 modules are presented in (Figures 4(c) and 4(d)). According to the enrichment scores, the corresponding relevant noncoding RNAs (ncRNAs) were closely associated with ubiquitin-mediated proteolysis module m1, spliceosome module m2, cell cycle modules m14 and m18, and endocytosis module m12, which included long noncoding RNAs (lncRNAs) *MALAT1* (modulelinks = 14, *P* = 7.6∗10^−3^), *FENDRR* (modulelinks = 18, *P* = 2.5∗10^−3^), *RNU1-1* (modulelinks = 23, *P* = 0), and *TUG1* (modulelinks = 17, *P* = 4.03∗10^−7^). The transcription factors (TFs) identified based on the enrichment scores were closely associated with GPR signaling pathway module m3, ECM-receptor interaction module m4, glutathione metabolism module m5, neuroactive ligand-receptor interaction module m9, endocytosis module m12, cell adhesion module m13, nucleotide excision repair module m17, homologous recombination module m16, and cell cycle modules m14 and m18, which included *E2F1* (modulelinks = 5, *P* = 3∗10^−4^), *TP53* (modulelinks = 6, *P* = 2∗10^−4^), *YBX1* (modulelinks = 4, *P* = 1.24∗10^−5^), *E2F4* (modulelinks = 3, *P* = 2∗10^−4^), *SP1* (modulelinks = 7, *P* = 4.3∗10^−3^), *BRCA1* (modulelinks = 3, *P* = 2.8∗10^−3^), *CREB1* (modulelinks = 5, *P* = 4.74∗10^−5^), and *CIITA* (modulelinks = 5, *P* = 4.19∗10^−7^). As shown in Table 5, these key genes, such as *MALAT1*, *RNU1-1*, *FENDRR*, *TUG1*, *E2F1*, *TP53*, *SP1*, *YBX1*, *BRCA1*, *E2F4*, *CREB1*, and *CIITA*, play significant functional roles in their associated modules, suggesting that these genes may play roles in cell cycle regulation, methylation, acetyltransferase activity, and the splicing cycle. According to the integrated analysis results, these key genes of lncRNAs and TFs might play pathogenic roles in the occurrence and progression of IPF.

### 3.5. Validation of the lncRNAs and TFs in IPF with qRT-PCR

Demographic and clinical features of the IPF patients and the healthy control group are listed in Table 6. Patients with IPF smoked fewer cigarettes than in the control group. Moreover, the clinical features of the IPF group were decreased in pulmonary function. To validate the results obtained through integrated analysis of the three datasets related to IPF, the relative expression of the key genes was analyzed by RT-PCR (Fig. S4). We found that 3 of the 4 candidate genes have statistically significant differences between the IPF and normal groups (*MALAT1*, *E2F1*, and *YBX1* with *P* < 0.01, *FENDRR* with *P* > 0.05).

## 4. Discussion

Chronic and progressive airway remodeling is a major characteristic of IPF with unknown etiology. Although accumulating evidence reveals that activated fibroblasts have important effects on the pathogenesis and progression of IPF, the underlying molecular mechanisms involved in the regulation of IPF remain unclear. Previous findings of gene regulation on IPF have mainly focused on protein-coding genes which can delay but do not inhabit the development of fibrosis. Recently, with the development of high-throughput sequencing technology, epigenetic researches provide new insights into the underlying molecular and etiological mechanisms of IPF. Epigenetics, such as functional ncRNAs, refers to heritable changes in DNA and chromatin that influence gene expression other than changes in DNA sequence and has gradually become the research hotspot. Multiple studies have indicated that lncRNAs can influence the pathological process involving the structural remodeling of pulmonary architecture and eventually lead to respiratory failure. As multifunctional adaptor molecules, lncRNAs play multifunctional roles in the regulation of gene expression by regulating mRNA decay, splicing, and gene looping by binding to DNA, proteins, and certain other RNAs [17, 18]. In this study, we integrated three publicly available microarray datasets (GSE2052, GSE44723, and GSE24206) and found that differential expression of 8483 genes comprised 988 upregulated and 7495 downregulated genes. Consistent with the results of previous studies on the molecular mechanism of IPF, we found that DEGs were mainly concentrated on the extracellular matrix and these biological functions were mainly related to cell adhesion, proliferation, cytoskeleton development, and cytokine interaction. After a series of bioinformatics analysis, the regulatory network consisting of key lncRNAs and transcription factors (TFs), which may contribute to the pathogenesis of IPF, was ultimately obtained. We found that the biological functions of these key genes, which were related to epithelial-mesenchymal transition (EMT), mainly focused on mitochondrial translation, RNA processing, and ubiquitin-mediated proteolysis. We performed a comprehensive literature search and judged by integrating degrees, closeness, and betweenness centrality of the regulatory network ultimately identifying 2 lncRNAs and 2 TFs (MALAT1, FENDRR and E2F1, YBX1, respectively). Subsequently, we further validate the expression levels of these key genes related to the regulation of pulmonary fibrosis in blood samples between the IPF and control groups using real-time polymerase chain reaction (RT-PCR). As a result, differential expression of these genes including downregulated YBX1 and upregulated MALAT1 and E2F1 reached statistical significance except for FENDRR between two groups.

The research on epithelial-to-mesenchymal transition (EMT) related to a fibrotic process has received an increased attention in recent years. Considering the possible false-negative or false-positive results between the individual studies and the research sample size, we integrated and analyzed the potential lncRNAs and TFs related to the pathogenesis and progression of IPF from the public microarray data. Metastasis-associated lung adenocarcinoma transcript 1 (MALAT1) located on chromosome 11q13.1, also known as nuclear-enriched abundant transcript 2 (NEAT2), is involved in various biological functions including molecular scaffolds for ribonucleoprotein complexes, transcriptional regulator for genes, and regulation of cell cycle [19]. Substantial research has confirmed that MALAT1 has many important physiological and pathological function in a wide range of diseases such as various solid cancers, septic lung injury, myocardial or renal ischemia-reperfusion injury, cardiac fibrosis, liver fibrosis, and silica-induced pulmonary fibrosis [20–23]. Furthermore, MALAT1 also play an important role in EMT related to the pulmonary fibrosis [24]. Although MALAT1 was reported to be mainly localized in the nucleus, it could transfer from the nucleus to the cytoplasm during the G2/M-phase cell cycle [25]. E2F1 located on chromosome 20q11.2, which was screened out and validated in this study, belongs to TFs of the nuclear factor of the E2F family and participates in the cell cycle G1/S phase regulation mediating both cell proliferation and apoptosis [26]. Many studies have confirmed that E2F1 could activate the expression of stromal markers related to EMT such as vimentin and fibronectin and facilitate the pathogenesis processes such as fibrosis and tumor progression [27]. YBX1 located on chromosome 1p34.2, which is another screened and validated transcription factor in this study, belongs to a member of cold-shock protein family and acts as an important regulator related to cell proliferation and cell cycle [28]. Some studies have reported a correlation between abnormal expression of YBX1 and EMT markers such as vimentin and N-cadherin [29]. The above results suggest that genes screened and validated in this study might act as key regulators of the pathogenesis and progression of IPF.

However, the limitations of this study are as follows. First, although key genes related to the pathogenesis of IPF have been screened and validated through integrating three datasets and performing a series of bioinformatics approaches, expression levels of these key genes need further validated experiments such as western blotting (WB) and immunohistochemistry analysis (IHC). Second, differential gene analysis is one of the crucial data analysis strategies for expression profiling of IPF in GEO datasets. However, the three datasets combined and analyzed in this study from the GEO database microarray and platform were not unified. Meanwhile, the sample sizes of these three datasets were relatively small and imbalanced. The potential selection bias and information bias were inevitable. Therefore, the accuracy and reliability of candidate genes could be improved greatly by integrating more various types of datasets. Third, verification of the expression levels of candidate genes in clinical samples is far from enough. Further functional verification of these candidate genes was necessary to perform by loss-of-function and gain-of-function experiments in vivo and in vitro. Lastly, the verification and discussion of the underlying molecular mechanisms of these candidate genes involved in the pathogenesis and progression of IPF will be necessary to confirm through a chromatin immunoprecipitation assay (CHIP) or dual-luciferase reporter gene assay and so on. Regardless of the limitations mentioned above, this study provided preliminary evidence for the candidate genes related to the pathogenesis of IPF. As a time-saving and cost-saving method for analysis of biomedical data, we took an extensive bioinformatics data-mining approach from different microarray platforms to obtain candidate lncRNAs and TFs in IPF. This study provided a framework and broad application prospects for exploring pathological molecular networks related to IPF.

## 5. Conclusion

This study provides reliable and comprehensive perspectives on the pathogenesis and progression of IPF; potential lncRNAs and TFs related to the pathogenesis of IPF were obtained through bioinformatics analysis. Ultimately, the 3 key genes that were found to show abnormal expression in IPF compared to normal lung tissues may be considered as biomarkers for the diagnosis and treatment of IPF, which should be verified in subsequent studies.

## Figures and Tables

**Table 1:** DEGs were identified from three datasets, including 262 upregulated and 191 downregulated genes in IPF compared with normal lung tissues (upregulated genes were defined with fold change (FC) > 0 as the cutoff criterion. The opposite was defined as downregulated genes).

DEGs	Gene names
Upregulated	SULF1, DEAF1, SCG5, DSG2, SLC1A4, CCND2, KCNN4, ST6GAL1, SLC38A1, SEC11C, XPOT, DPY30, PFKP, DDB1, HEPH, CXCL13, ATXN10, SEL1L3, CIAO1, CCL19, STARD5, SLFN12, ROR2, VAT1L, FPGT, GMPPA, COL18A1, COL7A1, PIGF, LMO4, FAM120A, SLC29A3, TCFL5, IGFBP2, UQCRQ, CCNA2, TWSG1, TCTN3, ASPN, PAM, BPIFB1, FAIM, FBLN2, SCARA3, COMP, ABCC5, DIO2, CHEK2, MCM4, TM9SF2, NAB1, DGKA, PTGFRN, FAT1, DOK5, CNIH1, ACTN1, PLA2G12A, MAGED1, ALG1, TWIST1, TRIM5, RCN2, CXCL14, ARMC1, STMN3, HMCN1, WDR5B, CROT, LEF1, TMEM14A, PLA2G4A, FKBP10, ABCC3, SPR, ROBO1, OXR1, CRLF1, TRIAP1, KDELR3, DIRAS3, BBS2, TGFB3, LGMN, CDK2AP1, CXCL12, RRM2, STRBP, TSPAN6, DAP, COL6A3, FZD6, TDO2, GMDS, PPP2R5E, SUPT7L, ZKSCAN7, CDKN3, CNTNAP1, IGF1, GSS, LRRC8D, TMEM98, TRIM2, LTBP1, BACE2, BRD8, COLEC11, FXN, PAFAH1B3, PGAM1, COL5A2, AOC1, ANTXR1, TMEM69, IMPACT, NET1, MXRA5, RCN1, MYO1E, DUSP23, CDH3, RHOD, CYP2S1, POSTN, ICMT, PDLIM4, C1QTNF6, ACVR1, SYTL2, PLA2G7, MFAP2, ZDHHC13, TMED10, ALDH1A3, SLN, CPOX, CDKN2C, PPIC, XRCC5, CLDN1, NSG1, ITGA7, R3HDM1, ERGIC2, TRIM36, EYA2, RPL39L, CCL13, RBP5, DONSON, SERPINB5, TXNDC15, HOMER3, ARL1, UBE2E3, CRYM, MEOX1, TMEM45A, COL15A1, ATP1B1, LDLRAD4, STEAP3, NABP2, BDKRB2, DCLK1, CFH, TRO, ECM1, PFN2, IL13RA2, MYOF, FHL2, CADPS, ITGAV, PCNA, PTK7, KIF2C, MEGF8, BMP4, PDCD2L, PRMT6, TP53BP1, OSBPL6, FMO1, PDE1A, PBX3, ELOVL4, ATF7IP, SYNDIG1, TMEM158, CFI, ALDH3A1, CKAP2, MRPL2, COL14A1, EGFL6, LHX6, THBS2, RRM1, YLPM1, TM7SF3, MLEC, CFB, BCL11A, GPR87, ZNF436, CLNS1A, ATIC, LGR4, CYP24A1, SEMA3C, PDGFC, TP63, ARMCX2, NUSAP1, ASB2, SLC39A6

Downregulated	MKLN1, ECHDC3, RAB32, SLC25A51, HOPX, MATN3, MAP2, ARHGAP6, EPB41L5, NRGN, HEY1, PCTP, ACVRL1, TBX5, ERMP1, NAGA, MPP1, TXNIP, LRRN3, FLOT1AATK, RCL1, CSF3R, ANXA3, TEK, GRK5, HES1, HSPA1L, GATA6, EMP2, SLCO2A1, PMM1, STARD13, SEC14L1, SPTBN1, GHRL, TSPAN7, NEBL, ZNF655, TMEM11, UIMC1, NCOA3, ZFP36, CREBBP, LDLR, RAB20, SERTAD1, PPFIBP1, REPS2, ELF1, CALCOCO2, CSRNP1, GPM6A, DLL4, FPR1, CARHSP1, ADARB1, LMO7, RCOR1, LRRC32, CTNNBIP1, CA4, PADI4, OSGIN1, CXCL2, EGFR, CHI3L2, LPIN2, ANKHD1, ARHGEF4, ARHGAP29, VAMP5, RAI2, CYTIP, PRX, IER5, DNM2, IL17RA, BHLHE40, SLC39A8, RAPGEF5, PTPRM, CNOT8, DLC1, TLK2, EPAS1, PRELP, MAFF, ABTB1, HSD17B6, NDEL1, HYAL1, HECA, HSPB8, DNAJB1, CDH13, RGS16, PTPN12, CD55, TIPARP, CRYAB, CD36, NUP153, PTPRB, ITSN2, TNNC1, MAPT, THBD, CDKN2D, AOC3, P2RY1, ZBTB16, CSF3, EDNRB, FAM167A, SRGAP2, SLCO1A2, DAPK2, AGTR1, RIMKLB, ASRGL1, ANG, CCK, BCL2L13, OSGIN2, ACSM5, KIAA0040, KDR, FUT1, DOCK9, GADD45G, CLDN5, LIFR, STXBP6, GPR4, S1PR1, SLC1A1, PLAG1, EDA, DENND3, IDI1, KHDRBS3, CLEC1A, INMT, MPP3, PLLP, MTSS1, FSTL3, CRTAC1, GTF2IRD1, F3, KLF10, KLRD1, FBLN5IZUMO4, PIR, MAOA, C1QL1, THRB, RNF182, ALDH6A1, FAM49A, ST6GALNAC3, SSFA2, SLC25A24, AMPH, ADAMTS8, PLSCR1, BCKDHA, STXBP4, FLRT3, AOX1, SYAP1, RLF, SSH2, DERA, PIM1, STARD3NL, SUN2, SEPP1, IL1R2, EIF2A, FAH, METTL7A, EIF4E3, CHRM2, 1-Mar, PDK1, TJP2, RASL11A, NKX3-1

**Table 2:** The significantly enriched analysis of differentially expressed genes in idiopathic pulmonary fibrosis.

Category	Pathway ID	Description	Count	*P* value
GOTERM_BP	GO:0007155	Cell adhesion	45	1.46327*E* − 09
GOTERM_BP	GO:0022610	Biological adhesion	45	1.50199*E* − 09
GOTERM_BP	GO:0042127	Regulation of cell proliferation	42	9.51714*E* − 07
GOTERM_BP	GO:0035295	Tube development	18	1.51015*E* − 05
GOTERM_BP	GO:0032103	Positive regulation of response to external stimulus	10	1.56397*E* − 05
GOTERM_BP	GO:0001501	Skeletal system development	22	1.77224*E* − 05
GOTERM_MF	GO:0005509	Calcium ion binding	38	0.000949643
GOTERM_MF	GO:0019955	Cytokine binding	10	0.001081524
GOTERM_MF	GO:0008009	Chemokine activity	6	0.004374834
GOTERM_MF	GO:0042802	Identical protein binding	27	0.00485805
GOTERM_MF	GO:0042379	Chemokine receptor binding	6	0.005747644
GOTERM_MF	GO:0008017	Microtubule binding	7	0.00692901
GOTERM_CC	GO:0044421	Extracellular region part	54	3.94172*E* − 10
GOTERM_CC	GO:0005615	Extracellular space	38	4.31806*E* − 07
GOTERM_CC	GO:0031012	Extracellular matrix	24	2.55104*E* − 06
GOTERM_CC	GO:0005578	Proteinaceous extracellular matrix	22	8.9433*E* − 06
GOTERM_CC	GO:0044459	Plasma membrane part	76	5.14236*E* − 05
GOTERM_CC	GO:0031226	Intrinsic to plasma membrane	48	0.000103242
KEGG_PATHWAY	hsa04512	ECM-receptor interaction	9	0.00286045
KEGG_PATHWAY	hsa04060	Cytokine-cytokine receptor interaction	17	0.003658523
KEGG_PATHWAY	hsa04510	Focal adhesion	13	0.01357663
KEGG_PATHWAY	hsa04610	Complement and coagulation cascades	7	0.014586106
KEGG_PATHWAY	hsa05414	Dilated cardiomyopathy	8	0.017081193
KEGG_PATHWAY	hsa00360	Phenylalanine metabolism	4	0.024804532

**Table 3:** The significant GO enrichment analysis of module gene function in idiopathic pulmonary fibrosis.

Category	Pathway ID	Pathway description	Count	*P* value
GO_BP_m1	GO:0000209	Protein polyubiquitination	47	1.77*E* − 64
GO_BP_m10	GO:0097711	Ciliary basal body-plasma membrane docking	32	6.55*E* − 71
GO_BP_m11	GO:0048193	Golgi vesicle transport	30	9.70*E* − 41
GO_BP_m12	GO:0006898	Receptor-mediated endocytosis	19	6.36*E* − 21
GO_BP_m13	GO:0060337	Type I interferon signaling pathway	20	1.37*E* − 38
GO_BP_m14	GO:0007059	Chromosome segregation	29	2.58*E* − 38
GO_BP_m15	GO:0009165	Nucleotide biosynthetic process	20	2.61*E* − 27
GO_BP_m16	GO:0036297	Interstrand cross-link repair	16	1.73*E* − 32
GO_BP_m17	GO:0006289	Nucleotide-excision repair	26	7.46*E* − 53
GO_BP_m18	GO:0000280	Nuclear division	24	7.63*E* − 35
GO_CC_m1	GO:0000151	Ubiquitin ligase complex	42	1.34*E* − 54
GO_CC_m10	GO:0044450	Microtubule organizing center part	19	1.93*E* − 29
GO_CC_m11	GO:0005875	Microtubule-associated complex	27	1.16*E* − 46
GO_CC_m12	GO:0030136	Clathrin-coated vesicle	22	8.55*E* − 33
GO_CC_m13	GO:0042611	MHC protein complex	5	6.87*E* − 10
GO_CC_m14	GO:0000775	Chromosome, centromeric region	35	4.74*E* − 59
GO_CC_m15	GO:0008074	Guanylate cyclase complex, soluble	2	2.57*E* − 06
GO_CC_m16	GO:0043240	Fanconi anemia nuclear complex	7	9.55*E* − 17
GO_CC_m17	GO:1990391	DNA repair complex	9	3.70*E* − 17
GO_CC_m18	GO:0005819	Spindle	17	5.08*E* − 23
GO_MF_m1	GO:0004842	Ubiquitin-protein transferase activity	69	5.10*E* − 99
GO_MF_m10	GO:0005200	Structural constituent of cytoskeleton	6	4.58*E* − 08
GO_MF_m11	GO:0003777	Microtubule motor activity	21	3.95*E* − 38
GO_MF_m12	GO:0030276	Clathrin binding	9	2.00*E* − 14
GO_MF_m13	GO:0042605	Peptide antigen binding	5	2.31*E* − 09
GO_MF_m14	GO:0043515	Kinetochore binding	4	9.27*E* − 10
GO_MF_m15	GO:0016776	Phosphotransferase activity	10	4.97*E* − 20
GO_MF_m16	GO:0140097	Catalytic activity, acting on DNA	13	5.26*E* − 17
GO_MF_m17	GO:0003684	Damaged DNA binding	16	9.67*E* − 31
GO_MF_m18	GO:0004674	Protein serine/threonine kinase activity	8	6.57*E* − 07

**Table 4:** The significant KEGG enrichment analysis of module gene function in idiopathic pulmonary fibrosis.

Pathway	Pathway description	Count	*P* value	Genes
KEGG_Pathway_m1	Ubiquitin mediated proteolysis	30	1.21*E* − 46	10273, 10477, 11065, 22954, 23327, 25898, 51433, 51434, 54926, 55070, 57154, 65264, 7318, 7320, 7321, 7323, 7326, 7328, 7332, 7428, 83737, 8454, 8697, 9021, 90293, 9246, 92912, 9820, 991, 6921
KEGG_Pathway_m2	Spliceosome	30	1.23*E* − 44	10084, 10262, 10285, 10594, 10907, 10946, 10992, 1665, 22827, 51340, 51690, 57187, 57819, 6428, 6429, 6430, 6432, 6434, 6625, 6626, 6628, 6635, 7307, 8175, 84321, 8449, 84991, 9775, 988, 9984
KEGG_Pathway_m15	Purine metabolism	26	8.05*E* − 39	10201, 11128, 124583, 171568, 205, 2987, 29922, 3704, 4831, 4832, 4833, 4881, 50484, 50808, 51251, 5138, 5315, 5422, 6240, 6241, 84284, 953, 955, 956, 2982, 2983
KEGG_Pathway_m16	Fanconi anemia pathway	20	2.23*E* − 37	2067, 2072, 2176, 2178, 2188, 2189, 22909, 29089, 51426, 5429, 55120, 5889, 6118, 6119, 672, 80010, 80198, 84464, 80233, 91442
KEGG_Pathway_m4	Protein digestion and absorption	21	1.16*E* − 36	1277, 1278, 1281, 1285, 1287, 1288, 1289, 1290, 1292, 1293, 1294, 1299, 1300, 1303, 1306, 1308, 255631, 7373, 80781, 81578, 85301
KEGG_Pathway_m5	Glutathione metabolism	20	1.44*E* − 36	124975, 2678, 2687, 27306, 2878, 2879, 2880, 2882, 2937, 2938, 2941, 2948, 2949, 2950, 2952, 373156, 4257, 4258, 493869, 51060
KEGG_Pathway_m6	Ribosome	20	4.77*E* − 35	11222, 29088, 51069, 51073, 51116, 51263, 51264, 51318, 54460, 54948, 6183, 63875, 63931, 64928, 64963, 64965, 64983, 65003, 65008, 79590
KEGG_Pathway_m17	Nucleotide excision repair	16	4.27*E* − 30	1022, 1069, 1642, 1643, 2067, 2072, 2968, 3978, 5111, 5425, 6118, 6119, 7507, 7508, 8451, 902
KEGG_Pathway_m9	Neuroactive ligand-receptor interaction	25	7.26*E* − 28	10161, 10800, 1131, 1241, 148, 154, 185, 1902, 1910, 2149, 2151, 2925, 3061, 3357, 3358, 4829, 4923, 5021, 5028, 5031, 624, 680, 6915, 7201, 9002
KEGG_Pathway_m3	Neuroactive ligand-receptor interaction	25	3.41*E* − 21	1129, 1268, 150, 152, 1813, 187, 1901, 1902, 1903, 2357, 2358, 2359, 2587, 2913, 2918, 4543, 4887, 59340, 624, 6752, 6755, 719, 728, 9294, 9568
KEGG_Pathway_m8	Ribosome biogenesis in eukaryotes	12	5.45*E* − 20	10171, 10799, 134430, 23560, 27341, 3692, 51096, 51119, 51602, 55341, 55651, 84916
KEGG_Pathway_m7	Ribosome	17	7.99*E* − 19	11224, 25873, 51065, 6128, 6141, 6156, 6157, 6160, 6168, 6169, 6181, 6201, 6205, 6229, 6231, 6232, 6234
KEGG_Pathway_m12	Endocytosis	16	3.91*E* − 16	10109, 1173, 1759, 1785, 1956, 22905, 26119, 27131, 273, 3949, 408, 5868, 6456, 6643, 867, 8976
KEGG_Pathway_m13	Herpes simplex infection	11	8.93*E* − 13	10379, 3105, 3113, 3115, 3122, 3134, 3434, 3661, 4938, 4940, 6041
KEGG_Pathway_m18	Cell cycle	7	9.18*E* − 11	4085, 701, 7272, 8379, 890, 891, 9133
KEGG_Pathway_m14	Oocyte meiosis	8	8.35*E* − 10	4085, 5516, 5525, 5528, 5529, 8379, 891, 9133
KEGG_Pathway_m11	Vasopressin-regulated water reabsorption	6	1.07*E* − 09	10540, 1639, 51164, 79659, 84516, 8655
KEGG_Pathway_m10	Pathogenic Escherichia coli infection	3	5.99*E* − 05	203068, 7277, 7846

**Table 5:** Functional roles of the strongest correlations of lncRNAs and TFs in IPF.

No.	Gene symbol	Full name	Function
1	MALAT1	Metastasis-associated lung adenocarcinoma transcript 1	Form molecular scaffolds for ribonucleoprotein complexes, acting as a transcriptional regulator for numerous genes, and involved in cell cycle regulation
2	RNU1-1	RNA, U1 small nuclear 1	Its related pathways are spliceosomal splicing cycle
3	FENDRR	FOXF1 adjacent noncoding developmental regulatory RNA	Bind to polycomb repressive complex 2 and/or TrxG/MLL complexes to promote the methylation of the promoters of target genes
4	TUG1	Taurine upregulated 1	Interacts with the polycomb repressor complex and functions in the epigenetic regulation of transcription, acting as a sponge for microRNAs
5	E2F1	E2F transcription factor 1	Bind preferentially to retinoblastoma protein pRB in a cell cycle-dependent manner and mediate both cell proliferation and p53-dependent/independent apoptosis
6	TP53	Tumor protein P53	Regulate expression of target genes, inducing cell cycle arrest, apoptosis, senescence, DNA repair, or changes in metabolism
7	SP1	Sp1 transcription factor	Be involved in many cellular processes, including cell differentiation, cell growth, apoptosis, immune responses, response to DNA damage, and chromatin remodeling
8	YBX1	Y-box binding protein 1	Be implicated in numerous cellular processes including regulation of transcription and translation, pre-mRNA splicing, DNA reparation, and mRNA packaging
9	BRCA1	Breast cancer type 1 susceptibility protein	Play a role in transcription, DNA repair of double-stranded breaks, and recombination which forms a large multisubunit protein complex known as the BRCA1-associated genome surveillance complex and interacts with histone deacetylase complexes
10	E2F4	E2F transcription factor 4	Act as proliferation-associated suppression genes and bind to all three of the tumor suppressor proteins pRB, p107, and p130
11	CREB1	CAMP responsive element binding protein 1	Its related pathways are development of HGF signaling pathway and circadian entrainment
12	CIITA	Class II major histocompatibility complex transactivator	Once it does not bind DNA but rather uses an intrinsic acetyltransferase activity to act in a coactivator-like fashion

**Table 6:** Demographic and clinical characteristics of the subjects.

	Healthy controls	IPF	*P* value
	*n* = 20	*n* = 20	
Age, mean (SD)	68.4 (4.7)	69.0 (6.6)	0.74
Gender, *n* (%)			0.07
Male	11 (55)	12 (60)	
Female	9 (45)	8 (40)	
Smoker, *n* (%)			0.001
Current	4 (20)	2 (10)	
Former	7 (35)	10 (50)	
Never	9 (45)	8 (40)	
Smoking dose (pack-year)	37 (14.5-58)	35.7 (12-56)	0.14
FVC (% predicted), mean (SD)	96.2 (11.7)	63.88 (17.0)	<0.01
DL_CO_ (% predicted), mean (SD)	80.8 (17.6)	42.04 (16.5)	<0.01
FEV1 (% predicted), mean (SD)	106.0 (18.6)	71.7 (11.0)	<0.001
FEV1/FVC (%), mean (SD)	79.9 (5.0)	57.8 (8.3)	<0.001

FVC: forced vital capacity; DL_CO_: diffusing capacity of carbon monoxide; FEV1: forced expiratory volume in one second.

## Data Availability

The datasets used and/or analyzed during the present study are available from the corresponding author on reasonable request.

## Supplementary Materials

Supplementary Materials Fig. S1: hierarchical clustering using differentially expressed genes across all samples from GSE2052. Fig. S2: hierarchical clustering using differentially expressed genes across all samples from GSE44723. Fig. S3: hierarchical clustering using differentially expressed genes across all samples from GSE24206. Fig. S4: the relative expression of the key genes was analyzed by RT-PCR. Table S1: sequences of primers for candidate genes. Table S2: the RT-PCR results of four candidate genes.
