# Supplementary material for: Integrated Analysis of a Gene Correlation Network Identifies Critical Regulation of Fibrosis by lncRNAs and TFs in Idiopathic Pulmonary Fibrosis
Source: Biomed Res Int. 2020 Jun 2;2020:6537462. doi: 10.1155/2020/6537462 (PMC7290873; doi:10.1155/2020/6537462)
Supplement: Supplementary Materials — Fig. S1: hierarchical clustering using differentially expressed genes across all samples from GSE2052. Fig. S2: hierarchical clustering using differentially expressed genes across all samples from GSE44723. Fig. S3: hierarchical clustering using differentially expressed genes across all samples from GSE24206. Fig. S4: the relative expression of the key genes was analyzed by RT-PCR. Table S1: sequences of primers for candidate genes. Table S2: the RT-PCR results of four candidate genes. [file 6537462.f1.pdf]

Fig. S1 Hierarchical clustering using differentially expressed genes across all samples from GSE2052.

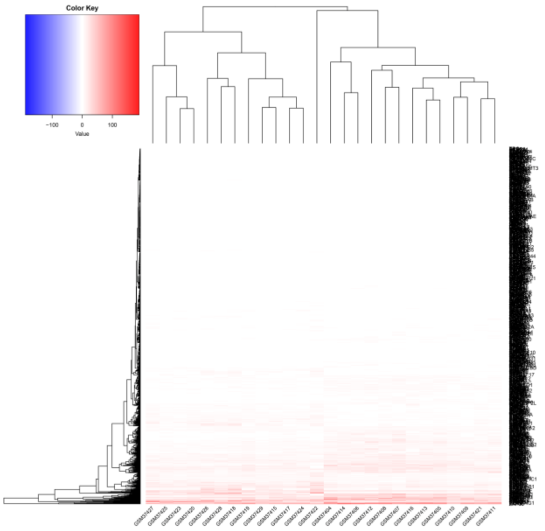

Fig. S2 Hierarchical clustering using differentially expressed genes across all samples from GSE44723.

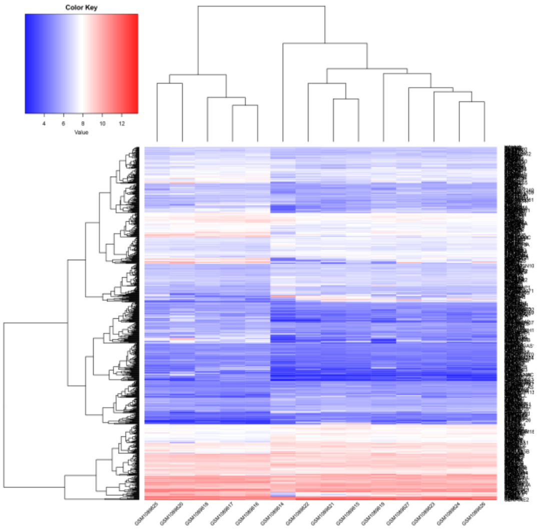

Fig. S3 Hierarchical clustering using differentially expressed genes across all samples from GSE24206.

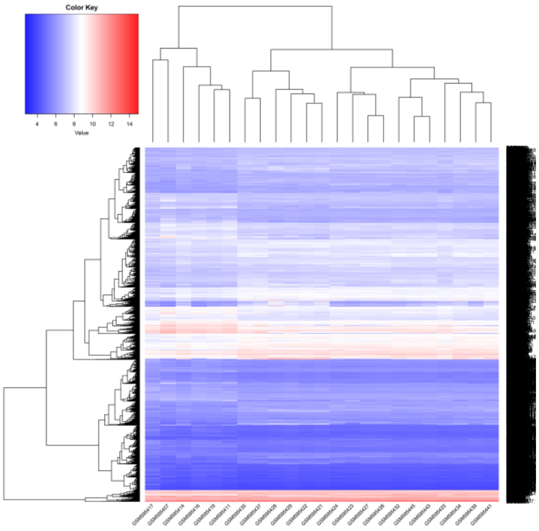

Fig. S4 The relative expression of the key genes was analyzed by RT-PCR.

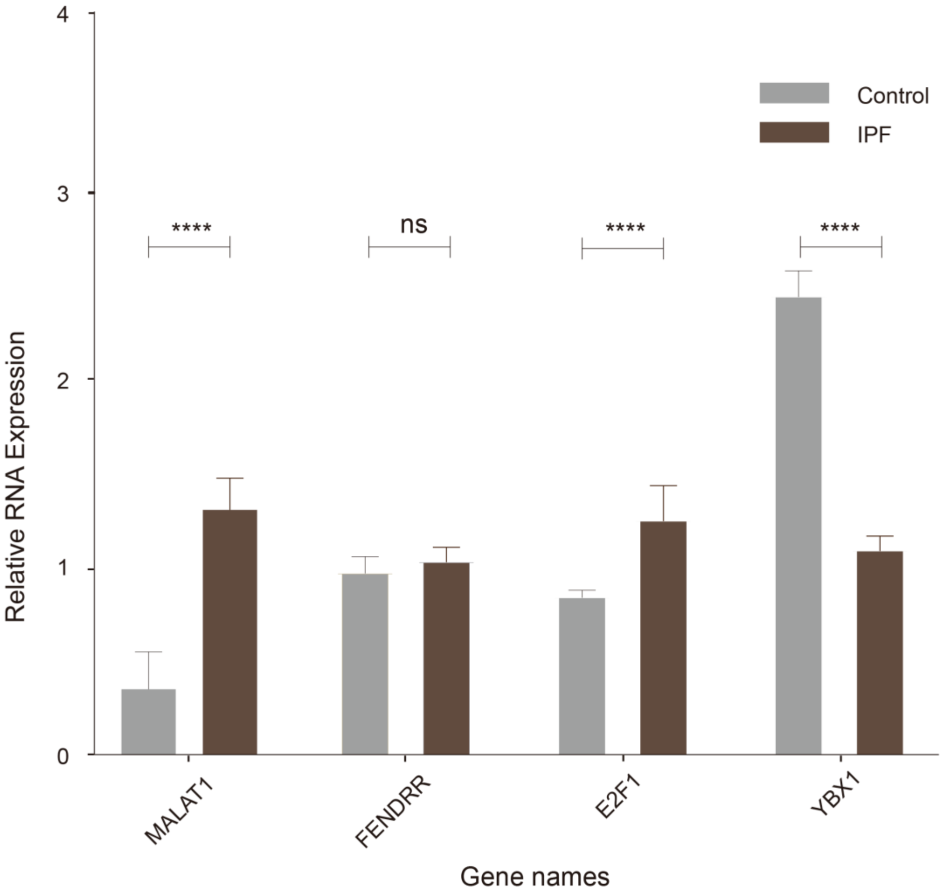

Supplementary Materials

Table S1. Sequences of primers for candidate genes

Gene	Primers (5'-3')	
	Forward	Reverse
MALAT1	AATGTTAAGAGAAGCCCAGGG-3	AAGGTCAAGAGAAGTGTCAGC
FENDRR	TAAAATTGCAGATCCTCCG	AACGTTCGCATTGGTTTAGC
E2F1	CACCATAGTGTCACCACCACCATC	TCTTGCTCCAGGCTGAGTAGAGAC
YBX1	ACAGAGCTGGACTGCGGTAT	TAGTAACCGGGTGCGGTAGA
GAPDH	CCTGCACCACCAACTGCTTAG	GTGGATGCAGGGATGATGTTC

Table S2. The RT-PCR results of four candidate genes

Gene name	group		P value
	IPF	Control	
MALAT1	1.31±0.17	0.35±0.20	P<0.001
FENDRR	1.05±0.08	0.99±0.09	P>0.05
E2F1	1.27±0.19	0.86±0.04	P<0.001
YBX1	1.11±0.08	2.47±0.14	P<0.001
